# Predictive Factors for Postoperative Pancreatic Fistula—A Swedish Nationwide Register-Based Study

**DOI:** 10.1007/s00268-020-05735-4

**Published:** 2020-08-20

**Authors:** C. Williamsson, K. Stenvall, J. Wennerblom, R. Andersson, B. Andersson, B. Tingstedt

**Affiliations:** 1 Department of Clinical Sciences Lund, Surgery, Lund University and Skåne University Hospital, Getingevägen 4, 221 85 Lund, Sweden; 2 Department of Surgery, Institute of Clinical Sciences, Sahlgrenska Academy, University of Gothenburg, Sahlgrenska University Hospital, Gothenburg, Sweden

## Abstract

**Background:**

A serious complication after pancreatoduodenectomy (PD) is postoperative pancreatic fistula (POPF). The aim of this study was to analyse the incidence and predictive factors for POPF by using a large nationwide cohort.

**Methods:**

Data from the Swedish National Registry for Pancreatic and Periampullary Cancer for all patients undergoing a PD from 2010 until 30th June 2018 were collected. The material was analysed in two groups, no POPF and clinically relevant (grade B and C) POPF.

**Results:**

A total of 2503 patients underwent PD, of which 245 (10%) developed POPF. Patients with POPF had significantly more overall complications (Clavien Dindo ≥3a, 75% vs. 21%, *p* < 0.001) and longer hospital stay (median 23 [16–35] vs. 11 [8–15], *p* < 0.001) than patients without POPF. The risk of POPF was higher with increased BMI (OR 1.08, *p* < 0.001). Preoperative presence of diabetes (OR 0.52, *p* = 0.012) and preoperative biliary drainage (OR 0.34, *p* < 0.001) reduced the risk of POPF. Reconstruction with pancreaticojejunostomy caused a more than two folded increase in POPF compared with pancreaticogastrostomy (OR 2.41, *p* < 0.001). Weight gain ≥2 kg on postoperative day 1 was also a risk factor (OR 1.76, *p* < 0.001).

**Conclusion:**

A high BMI, a pancreaticojejunostomy and postoperative weight gain were risk factors for developing POPF. Diabetes or preoperative biliary drainage was protective.

## Introduction

Postoperative pancreatic fistula (POPF) is one of the most harmful complications after pancreatoduodenectomy (PD), and it is considered to be the main underlying factor for downstream morbidity. The International Study Group of Pancreatic Surgery has established an international definition for clinically relevant POPF [1], which allows comparisons between institutions and countries.

There are many reports on different predisposing factors for POPF, which can be divided in patient, tumour and operating factors. Obesity, increased operative time, intraoperative blood loss and transfusions and a soft parenchyma are some documented risk factors [2–4]. Based on these risk factors, predictive risk scores have emerged [5, 6].

The efforts of the last decade to improve the perioperative care by creating high-volume units, reforming management of complications, and introducing enhanced recovery programmes have decreased mortality and improved the outcome [7, 8]. The overall rate of POPF does however remain unaffected [9].

Sweden has a population of approximately 10 million inhabitants. During the last 15 years, pancreatic resections were gradually centralized. Since 2015 pancreatic surgery is only performed at six regional university centres in Sweden. Representatives from these centres convened to establish a national registry for pancreatic tumours and operations, and in 2009 the Swedish National Registry for Pancreatic and Periampullary Cancer was introduced. It has until now enrolled a total of 13,800 patients [10].

The aim of this study was to analyse the incidence of and predictive factors for POPF after PD, based on the Swedish national registry.

## Methods

Data were retrieved from the Swedish National Registry for Pancreatic and Periampullary Cancer. The registry contains prospectively registered data on patients with pancreatic or periampullary lesions, as well as all patients undergoing pancreatic surgery. It is a nationwide secure web-based registry. The coverage rate has been over 90% for all patients who had an operation, resected or not, since 2010. The registry was validated in 2016 [11].

In Sweden, pancreatic resections are centralized, and in 2015, 93% of the resections were performed by one of the six regional centres [11]. All of these centres are high volume centres and all PDs are performed by high volume consultant surgeons. The annual PDs are >150 at one site, 75–150 at four centres and 50 at one. Two of these centres uses pancreaticogastrostomy (PG) as standard method of reconstruction, while the rest perform a pancreaticojejunostomy (PJ), of which about 90% is duct to mucosa. Traditionally, patients in Sweden are accepted for PD due to a suspicion of periampullary malignant tumour, and not symptomatic relief of chronic pancreatitis. The proportion of patients who underwent PD with a benign histopathology was 15% in 2018 [10].

In this study, patients with periampullary tumours undergoing PD from 1 January 2010 until 30 June 2018 were included. Patient-related factors such as sex, age at diagnosis, BMI (body mass index), weight loss (≥10% of body weight) and comorbidity were collected, as well as tumour location and if preoperative biliary drainage or neoadjuvant therapy was used. Furthermore, intraoperative and postoperative variables were obtained. Intraoperative factors included blood loss, transfusion, operation time, vascular resection and method of reconstruction. The postoperative outcome was extracted and classified according to international standards; Clavien–Dindo classification [12], deep infection (abscess), postpancreatectomy haemorrhage (PPH) [13], delayed gastric emptying (DGE) [14], bile leakage, reoperation, and 90-day mortality. Clavien–Dindo grades ≥3a was considered as major complications, based on the need for intervention. The PPH and DGE grades were merged to one event, present or not, since these complications have been recorded differently during the years in the registry. The international gold standard definition of POPF was used, where only the clinically relevant fistulas, i.e. grades B and C, were defined as fistulas [1]. BMI was defined according to the WHO classification. Postoperative weight gain was defined as ≥2 kg on postoperative day 1, based on previous data showing that a 2 L intraoperative positive fluid balance may increase the risk of complications [15]. All patient data from the register were individually reviewed for drain amylase output in relation to number of days with drain, Clavien–Dindo score, postoperative outcome, interventions and hospital stay, to grade the pancreatic fistula according to the latest POPF definition. The biochemical leaks were considered as “no POPF”. All data were sorted and analysed in two groups, “no POPF” or presence of “POPF”.

The study protocol was approved by the Human Ethics Committee at Lund University, Dnr 2018/783.

### Statistics

Descriptive data are presented as numbers and percentages, and median and interquartile range, as appropriate. Differences between the groups were evaluated by the Chi-square analysis for categorical variables and Mann–Whitney *U*-test for continuous variables. Multivariable analysis was performed using a stepwise backward regression of the variables with *p* < 0.25 in the univariable analysis. OR > 1 imply a higher risk for POPF. A *p* value of <0.05 was considered significant. Statistical analysis was conducted using SPSS^®^ version 25.0.0.2 (SPSS Inc^®^, Chicago, IL, USA).

## Results

### Patient and tumour characteristics

In total, 2503 patients underwent PD due to a periampullary tumour during the study period. There were 1344 (54%) men, and the median age was 68 (IQR 62–74) years, with no different distribution among the patient with or without POPF. A clinically relevant POPF affected 245 (10%) patients. The patients with a POPF had a significantly higher BMI and lesser proportion of diabetes, weight loss and preoperative biliary drainage than the group without POPF, see Table 1. The distribution of tumour location is presented in Table 2. A tumour located in the duodenum or distal bile duct was associated with a higher risk of POPF, while a tumour in the pancreatic head was accompanied by a significantly decreased risk of POPF.

**Table 1:** Demographics for patients with and without POPF

	*n*	Total (*n* = 2503)	No POPF (*n* = 2258)	POPF (*n* = 245)	*p* value
Male sex	2503	1344 (54)	1202 (53)	142 (58)	0.159*
Age (years)	2503	68 (62–74)	68 (62–74)	68 (62–73)	0.485^#^
BMI	2405	24.7 (22.3–27.7)	24.6 (22.3–27.4)	26.3 (23.5–29.9)	**<0.001**^#^
Smoking	2399	423 (18)	380 (17)	43 (19)	0.704*
Heart disease	2468	793 (32)	704 (32)	89 (37)	0.126*
Diabetes	2481	484 (20)	455 (20)	29 (12)	0.002*
Weight loss	2458	1279 (52)	1188 (54)	91 (37)	<0.001*
Preop biliary drainage	2479	1570 (63)	1472 (66)	98 (40)	<0.001*
Neoadjuvant treatment	2494	70 (3)	64 (3)	6 (3)	0.729*
ASA ≥ 3	2478	610 (25)	542 (24)	68 (28)	0.142*

Data presented as median (IQR) or numbers (%)

Bold values indicate statistical significance *p* < 0.05

*BMI* body mass index, *ASA* physical status classification system by the American Society of Anesthesiologists

*Chi-square, ^#^Mann–Whitney *U*-test

**Table 2:** Tumour location in relation to presence of POPF

	Total (*n* = 2500)	No POPF (*n* = 2257)	POPF (*n* = 243)	*p* value
Duodenum	204 (8)	172 (8)	32 (13)	0.003*
Distal bile duct	361 (14)	302 (13)	59 (24)	<0.001*
Ampulla vateri	312 (12)	273 (12)	39 (16)	0.085*
Head of pancreas	1561 (62)	1452 (64)	109 (45)	<0.001*
Others (*n* = 66)	62 (2)	58 (3)	4 (2)	0.371*

Data presented as actual numbers within each tumour location and % as proportion of each POPF-group

Bold values indicate statistical significance *p* < 0.05

*Chi-squared

### Intraoperative data

The duration of the operation, intraoperative blood loss or intraoperative transfusion were not correlated to POPF. There were significantly less vascular resections amongst patients who developed POPF. PJ was typically the choice of reconstruction (*n* = 1767, 71%) and was associated with a higher risk of POPF than PG, as given in Table 3. Somatostatin was used prophylactic in 441 of 723 (63%) PGs and 452 of 1767 (27%) PJs.

**Table 3:** Intraoperative data for patients with or without POPF

	*N*	Total (*n* = 2503)	No POPF (*n* = 2258)	POPF (*n* = 245)	*p* value
Operative time (min)	2484	390 (329–451)	390 (329–451)	389 (325–454)	0.858^#^
Vascular resection	2503	459 (18)	440 (20)	19 (8)	<0.001*
Intraoperative blood loss (ml)	2503	500 (300–900)	500 (300–900)	500 (350–1000)	0.332^#^
Intraoperative transfusion	2500	485 (19)	444 (20)	41 (17)	0.267*
Pancreaticojejunostomy	2490	1767 (71)	1560 (70)	207 (85)	<0.001*

Data presented as median (IQR) or numbers (%)

Bold values indicate statistical significance *p* < 0.05

*Chi-squared, ^#^Mann–Whitney *U*-test

### Postoperative outcome

The postoperative outcome for the two groups is listed in Table 4. All individual complications, except biliary leakage, were more common in the group with POPF. A surgical complication affected a total of 1172 (47%) patients, divided into DGE, surgical infection, biliary leakage, PPH and reoperation.

**Table 4:** Postoperative outcome in relation to POPF

	*N*	Total (*n* = 2503)	No POPF (*n* = 2258)	POPF (*n* = 245)	*p* value
Weight gain POD1 (≥2 kg)	1799	1121 (62)	981 (61)	140 (74)	0.001*
Drain (days)	2300	6 (4–9)	6 (4–8)	10 (6–19)	**<0.001**^#^
Somatostatin^a^	2418	894 (36)	822 (38)	72 (30)	0.011*
Clavien Dindo ≥3a	2328	666 (27)	482 (21)	184 (75)	<0.001*
Medical complication	2503	527 (21)	430 (19)	97 (40)	<0.001*
Surgical complication	2325	1172 (47)	927 (45)	245 (100)	<0.001*
If surgical complication = yes	1172				
DGE		470 (19)	348 (15)	122 (50)	<0.001*
Surgical infection		424 (17)	297 (13)	127 (52)	<0.001*
Intraabdominal abscess		259 (10)	145 (6)	114 (47)	<0.001*
Biliary leakage		110 (4)	90 (4)	20 (8)	0.461*
PPH		211 (8)	131 (6)	80 (33)	<0.001*
Reoperation		243 (10)	142 (6)	101 (41)	<0.001*
Length of stay (days)	2305	12 (8–17)	11 (8–15)	23 (16–35)	**<0.001**^**#**^
90-day mortality	2503	85 (3)	56 (3)	29 (12)	<0.001*

Data presented as median (IQR) or numbers (% of total in respective group)

Bold values indicate statistical significance *p* < 0.05

*Chi-squared, ^#^Mann–Whitney *U*-test

^a^Prophylactic somatostatin DGE delayed gastric emptying, *PPH* postpancreatectomy haemorrhage, *POD1* postoperative day 1

The logistic regression showed that the only preoperative independent risk factor associated with POPF was increased BMI, while presence of diabetes or preoperative biliary drainage was protective against POPF, as given in Table 5. Reconstruction with PJ was independently related to increased risk for POPF, as well as weight gain ≥2 kg on postoperative day 1. Somatostatin was not a protective factor for POPF, whereas a vascular resection was.

**Table 5:** Univariable and multivariable analysis to identify predictive factors for POPF

	Event/total	Univariable analysis			Multivariable analysis		
		OR	95% CI	*p* value	OR	95% CI	*p* value
Sex (male)	1344/2503	1.21	0.93–1.58	0.159	1.34	0.96–1.86	0.089
BMI	2405/2503	1.09	1.06–1.12	<0.001	1.08	1.05–1.12	**<0.001**
Heart disease	793/2468	1.24	0.94–1.63	0.126	1.29	0.91–1.84	0.150
Diabetes	484/2481	0.53	0.35–0.79	0.002	0.52	0.31–0.87	**0.012**
Weight loss	1279/2458	0.51	0.39–0.67	<0.001	0.76	0.54–1.08	0.123
Preoperative biliary drain	1570/2479	0.35	0.27–0.46	<0.001	0.34	0.25–0.48	**<0.001**
ASA ≥ 3	610/2478	1.21	0.90–1.62	0.215	1.12	0.77–1.64	0.560
Vein resection	459/2503	0.35	0.22–0.56	<0.001	0.45	0.25–0.82	**0.009**
Pancreatic anastomosis (PJ)	1767/2490	2.39	1.67–3.42	<0.001	2.41	1.51–3.85	**<0.001**
Somatostatin	894/2418	0.69	0.52–0.92	0.011	0.86	0.59–1.25	0.425
Weight gain ≥2 kg POD 1	1121/1799	1.79	1.28–2.51	0.001	1.76	1.22–2.54	**0.002**
Location (pancreatic head)	1561/2503	0.45	0.34–0.58	<0.001	0.48	0.34–0.67	**<0.001**

Bold values indicate statistical significance *p* < 0.05

*BMI* Body Mass Index, *PJ* pancreaticojejunostomy, *POD* postoperative day

## Discussion

This study from the Swedish national registry of resected periampullary tumours shows that a reconstruction with PJ and postoperative weight gain of 2 kg or more is accompanied by POPF to a higher degree. Furthermore, presence of diabetes and preoperative biliary drainage is protective as well as a tumour location in the pancreatic head and the intraoperative need for vein resection.

POPF is the most harmful complication after PD and correlates to other complications, hospital stay, mortality and increased costs [16, 17]. Despite advantages in surgical technique, perioperative care, centralization and introduction of enhanced recovery programme, the incidence of POPF remains unaltered [9]. The incidence of POPF of 10% in this Swedish study is in concordance with or even below international figures, 11.1–16.7% [18–20].

Most of the findings in this study are not controversial and support previously published papers. Presence of diabetes and a preoperative biliary drainage are features described earlier related to a reduced risk of POPF [20, 21] as well as tumour location and its correlation to POPF [4, 18, 22]. These are parameters associated with inflammation of the parenchyma or obstruction of the pancreatic duct, leading to pancreatic firmness. For instance, Mathur et al. [23] showed on surgical specimens, that total pancreatic fat was significantly decreased and fibrosis was increased in patients with diabetes. Furthermore, in this study the tumours in the duodenum and distal bile duct were most prone for POPF.

Most of the different risk factors identified here can be found in the most commonly accepted and used fistula risk scores by Roberts, Callery and colleagues [5, 6]. With a preoperative evaluation of the risk of POPF, the surgical consultation could be individualized to the patient, providing the possibility to alter the clinical pathway or avoid postoperative abdominal drainage for low risk patients, to minimize postoperative deep abscesses [24, 25].

Obese patients have an increased risk of overall complications after PD, and several studies have shown an increased risk for POPF specifically [2, 20, 21, 26]. Here, median BMI was higher for the group with POPF, and higher BMI was independently correlated with POPF. Overweight and obese patients have a softer pancreatic gland due to fatty infiltration. The pancreatic texture is known to influence the risk for POPF, and a soft gland results in a higher frequency of POPF [26].

Ellis et al. [20] demonstrated in a large cohort of 15,000 PDs, that patients not receiving neoadjuvant treatment had an increased risk of POPF. This was not supported by the Swedish data which could be explained by the low number of just 70 (3%) Swedish patients receiving neoadjuvant chemotherapy, or that radiation therapy never is used in the neoadjuvant setting in Sweden.

There are different conclusions in the literature regarding intraoperative blood loss and its correlation with POPF. In the fistula risk score (FRS) by Callery et al. [6] it is considered a risk factor with increasing blood loss, while others have shown no correlation [26]. An alternative, and updated, FRS (ua-FRS) has been evolved, excluding intraoperative blood loss and including male sex, which is recently validated for both open and minimally invasive PD [27]. In this study from the Swedish registry, intraoperative blood loss was not associated with POPF. Further, male sex was not a significant predictor for POPF, to the contrary of ua-FRS.

Vascular resection was shown to be protective for POPF in this study. This is in concordance with Yamamoto et al. [28] who demonstrated an almost four folded risk increase if the tumour “was away from the portal vein”. Likewise, when comparing PD with and without vein resection it has been shown that the group with vein resection have lower frequency of POPF [29, 30]. The result might be influenced by neoadjuvant treatment or pertain to the same model of explanation as tumour location, with a large tumour in need of vascular resection to be more likely to obstruct the pancreatic duct and increase the firmness of the gland.

A weight gain of ≥2 kg on POD 1 was a significant predictor for POPF in this study. Postoperative fluid overload is previously shown to give adverse outcome [31]. Goal directed fluid therapy is therefore important and included in most enhanced recovery programs [32]. Since the registry does not offer data on intraoperative fluid administration, it cannot be ruled out that postoperative weight gain in fact is a sign of early pancreatic leak.

PJ was significantly associated with a more than two folded increase in POPF compared with PG. The technique of pancreaticoenteric anastomoses has been widely debated, and none has convincingly been proven better than the other, according to the International Study Group of Pancreatic Surgery [33]. There are some randomized controlled trials comparing PG and PJ, of which some suggest PG to be superior to PJ (8–11% vs. 20–33%) regarding the rate of clinically relevant POPF [34, 35]. On the other hand, several studies fail to find any difference in POPF rate at all, including a recent Cochrane analysis [36–40]. Somatostatin was more commonly used with PGs in this study. It was however not independently correlated with POPF in the multivariable analysis.

The reason for the substantial difference in the Swedish data is not clear. Even though the regional centres perform different anastomoses, the perioperative care is otherwise greatly harmonized due to the collaboration of the Swedish registry. All PDs are performed at high volume centres, by high volume surgeons. Even though there are not clear evidence to support one pancreatic anastomose over the other, it has been argued that a standardized and consistent practise of a single technique might reduce POPF rate [41].

The major strength of this study is the large sample size and the national population-based coverage of several high-volume units, with only high-volume surgeons. This study has limitations inherent to the retrospective analysis of registry data. Even though data in the registry is continuously registered, some variables have changed over the years and from the available variables, manual assessments were needed to create new variables. Despite the registry is validated, one might question the accuracy of data input. Moreover, the registry does not contain information on pancreatic duct size or intraoperative assessment on pancreatic texture.

In conclusion, a higher BMI, pancreaticojejunostomy and postoperative weight gain were predictive factors for developing POPF in this study. Diabetes, preoperative biliary drainage and a tumour located in the head of pancreas in need for vascular resection were protective and are associated with a firm pancreatic tissue. The only modifiable factors identified are the method of reconstruction and postoperative weight gain.
